# Impact of Sinogram Affirmed Iterative Reconstruction (SAFIRE) Algorithm on Image Quality with 70 kVp-Tube-Voltage Dual-Source CT Angiography in Children with Congenital Heart Disease

**DOI:** 10.1371/journal.pone.0091123

**Published:** 2014-03-10

**Authors:** Pei Nie, Haiou Li, Yanhua Duan, Ximing Wang, Xiaopeng Ji, Zhaoping Cheng, Anbiao Wang, Jiuhong Chen

**Affiliations:** 1 Shandong Provincial Key Laboratory of Diagnosis and Treatment of Cardio-Cerebral Vascular Diseases, Shandong Medical Imaging Research Institute, Shandong University, Jinan, Shandong, China; 2 Department of Cardiovascular Surgery, Shandong Provincial Hospital, Jinan, Shandong, China; 3 CT Research Collaboration, Siemens Ltd. China, Beijing, China; Centro Nacional de Biotecnologia (CNB-CSIC), Spain

## Abstract

**Purpose:**

To compare the image quality and diagnostic accuracy between sinogram affirmed iterative reconstruction (SAFIRE) algorithm and filtered back projection (FBP) reconstruction algorithm at 70 kVp-tube-voltage DSCT angiography in children with congenital heart disease (CHD).

**Materials and Methods:**

Twenty-eight patients (mean age: 13 months; range: 2–48 months; male: 16; female: 12; mean weight: 8 kg) with CHD underwent 70 kVp DSCT angiography. Imaging data were reconstructed with both FBP and SAFIRE algorithms. Subjective image quality was evaluated on a five-point scale. The parameters of image noise, signal-to-noise ratio (SNR) and contrast-to-noise ratio (CNR) on the objective image quality were compared for the two reconstruction algorithms. Surgery was performed in 20 patients, whereas conventional cardiac angiography (CCA) was performed in 8 patients. The diagnostic accuracy was evaluated on the surgical and/or CCA findings. The effective radiation doses were calculated.

**Results:**

Compared to FBP algorithm, SAFIRE algorithm had significantly higher scores for subjective image quality (P<0.05), and lower image noise (P<0.05) as well as higher SNR &CNR values (P<0.05). There was no significant difference in the diagnostic accuracy between the FBP and SAFIRE algorithm (χ^2^ = 1.793, P>0.05). The mean effective dose for 70 kVp DSCT angiography was 0.30±0.13 mSv.

**Conclusions:**

The SAFIRE algorithm can significantly reduce image noise and improve the image quality at 70 kVp DSCT angiography for the assessment of CHD in children.

## Introduction

The recent innovations in multi-detector CT technology with improved spatial and temporal resolution have extended the application of cardiovascular imaging in the assessment of congenital heart disease (CHD) in children [1]–[6]. Radiation exposure delivered by CT is now of particular concern, especially in paediatric population. In contrast to adults, infants and children are more radiosensitive and have a longer life span to potentially develop radiation-induced carcinogenesis. The “ALARA” (as low as reasonable achievable) principle has to be considered thoroughly before each examination for children. Several dose reduction strategies, such as reduced tube voltage, automated tube current modulation, weight-adjusted tube current, minimization of z-axis coverage as well as use of the prospective ECG-triggering sequential mode and high-pitch mode, have been successfully implemented into paediatric cardiac CT angiography and have been shown to effectively lower the radiation dose [1]–[3], [4]–[8].

Recently, the second generation DSCT system (Definition Flash, Siemens Healthcare, Forchheim, Germany) provides a new x-ray tube with a tube voltage of 70 kVp. Lowering the tube voltage can reduce the radiation dose and effectively increase the contrast enhancement [9]–[11]. Using iodine as contrast agent, it is shown that the fraction of photons in the energy range around the k-edge of iodine is the highest for a tube voltage of about 63 kVp. Therefore, a tube voltage between 60 kVp and 70 kVp is the best choice for CT angiography [12]. Although the 80 kVp setting is now routinely used for infants and small children CT examinations [3], [4], using 70 kVp instead of 80 kVp should shift the mean photon energy of the x-ray beam closer to the k-edge of iodine and therefore improve the contrast between vessels and the surroundings. The downside of the low-tube-voltage technique is an increase in the image noise, which can be compensated by increasing the tube current and using the iterative reconstruction (IR) algorithm [13]. Gnant R et al. [14] demonstrated that neck CT at 70 kVp was feasible with 34% dose reduction compared to the standard protocol at 120 kVp.

Iterative reconstruction (IR) algorithms have been introduced to help reduce the quantum noise associated with the traditional filtered back projection (FBP) reconstruction algorithm. Recent studies have shown that IR algorithms, such as iterative reconstruction in image space (IRIS; Siemens), iDose (Philips), adaptive statistical iterative reconstruction (ASIR; GE Healthcare) and model-based iterative reconstruction (MBIR; GE Healthcare) etc. can improve the image quality and reduce image noise as well as radiation dose in comparison with FBP reconstruction algorithm [15]–[21]. Recently, a new IR algorithm- sinogram affirmed iterative reconstruction (SAFIRE; Siemens) has been introduced into clinical use. SAFIRE is a raw-data-based IR algorithm which compares reconstructed and measured data in the raw data domain and iteratively corrects the images [22]–[27]. Several studies have shown the benefit of SAFIRE algorithm for various clinical applications, including chest [22] and abdominal CT [23], as well as cardiac [24]–[26] and body CT angiography [27] with the improved image quality and image noise reduction which contributes to the radiation dose saving.

To the best of our knowledge, this is the first study to address the 70 kVp-tube-voltage combined with SAFIRE algorithm for paediatric cardiac DSCT angiography. The purpose of this study was to compare the image quality and diagnostic accuracy of 70 kVp-tube-voltage DSCT angiography in children with CHD by two different reconstruction techniques of sinogram affirmed iterative reconstruction (SAFIRE) algorithm and filtered back projection (FBP) reconstruction algorithm.

## Materials and Methods

### Patients

Our study received the approval from Shandong Medical Imaging Research Institute Ethics Board and written informed consent was obtained from the parents of all patients. In our institution, DSCT angiography is part of the cardiovascular assessment in patients with CHD besides transthoracic echocardiography.

Between January and October 2013, 34 consecutive patients with CHD referred for DSCT examinations were enrolled in this study. Exclusion criteria were nephropathy (n = 2) and hypersensitivity to iodinated contrast (n = 4). A total of 28 patients have been included in this study. All anomalies were confirmed by the surgical and/or the conventional cardiac angiography (CCA) findings. Surgery was performed in 20 patients, and CCA was performed in 8 patients.

### DSCT protocol

All examinations were performed on a second generation DSCT scanner (Somatom Definition Flash, Siemens Healthcare, Forchheim, Germany). Short-term sedation was achieved with oral administration of chloral hydrate. All patients were free-breathing. The scan range was from the bottom of the heart to the thoracic inlet in a caudocranial direction.

Scanning parameters were as follows: 0.28 s gantry rotation time, 2×64×0.6 mm detector collimation, a slice collimation 2×128×0.6 mm by z-flying focal spot technique, 70 kV tube voltage and weight adapted setting for tube current (70–80 mAs/rotation for patients <5 kg body weight, 80–100 mAs/rotation for patients 5–10 kg body weight, 100–130 mAs/rotation for patients >10 kg body weight). In patients with a relatively regular heart rate, prospective ECG-triggered high-pitch spiral scan mode was used with a pitch of 3.4. Data was acquired at 10% of R-R interval in order to obtain a systolic acquisition window for the proximal segments of coronary arteries. In patients with an irregular heart rate, prospective ECG-triggering sequential scan mode was chosen. The acquisition window was set at 40%–40% of R-R interval.

Iodinated contrast medium (Schering Ultravist, Iopromide, 350 mg I/ml, Berlin, Germany) was injected via peripheral veins using a double-head power injector (Stellant; Medrad, Indianola, PA, USA) with a volume of 1.5 ml/kg body weight followed by a saline chaser of 1.0 ml/kg body weight. The interval from injection to data acquisition was set at 25 seconds. Injection rate was calculated with the total injected volume divided by 25 s. For example, a 6-kg baby would be injected with 9 ml of contrast medium and 6 ml saline at flow rate of 0.6 ml/s.

### Image reconstruction

All datasets were reconstructed with both FBP and SAFIRE algorithms with a slice thickness of 0.75 mm and an increment of 0.5 mm. In SAFIRE algorithm, 5 presets (strength 1–5) can be adjusted for the level of noise reduction, 1 being the weakest and 5 being the strongest [22]. As recommended by the manufacturer, a medium strength of 3 was applied in all patients in this study. The medium smooth-tissue convolution kernel B26f and I26f were used in FBP and SAFIRE algorithms, respectively.

### Image quality analysis

All images were assessed on the subjective and objective image quality with a MMWP (Multiple Modality Workplace, Siemen Healthcare, Forchheim, Germany). Images reconstructed with SAFIRE and FBP algorithms respectively were reviewed in random order. Two radiologists specified on cardiac imaging with more than 5 years' experience independently interpreted the image quality on axial, multiplanar reformation (MPR), maximum intensity projection (MIP) and volume rendering (VR) images.

Subjective image quality analysis regarding to graininess, sharpness and overall image quality was assessed using a 5-grade scoring system (5, excellent; 4, good; 3, adequate; 2, limited diagnostic value; 1, uninterpretable) [28]. For any disagreement between the two observers, consensus agreement was achieved.

The image noise, signal-to-noise ratio (SNR) and contrast-to-noise ratio (CNR) were evaluated as objective image quality parameters. The measurement was done on the 0.75 mm-thick axial images by one observer who was not involved in the subjective image quality evaluation. Regions of interest (ROIs) were drawn in the ascending aorta and the pulmonary trunk as large as the diameter of the lumen, carefully avoiding the vessel wall. The image noise was defined as the standard deviation of the attenuation value and the SNR and CNR were calculated according to the following equations: SNR =  mean_vessel/SD_fat and CNR =  (mean_vessel- mean_fat)/SD_fat, respectively, where mean_vessel is the mean CT value of the vessel, mean_fat is the mean CT value of the perivascular fat and SD is the standard deviation of ROI.

### Diagnostic performance analysis

Blinded to the results of surgical and/or CCA findings, two radiologists who were not involved in image quality assessment evaluated all images in consensus. Both FBP and SAFIRE series were presented in random order. Using surgical and/or CCA findings as the reference standard, the diagnostic accuracy was calculated compared between FBP and SAFIRE algorithms.

### Radiation dose estimations

The volume CT dose index (CTDIvol) and dose-length product (DLP) were obtained from the CT system after each examination. The effective radiation dose (mSv) was calculated from the DLP (mGy·cm) multiplied by 2 to adapt it to the 16-cm phantom (the DLP for the body surface area was given for a 32-cm phantom on the scan protocol). The corrected DLP value was then multiplied by the infant-specific conversion coefficients given for a 16-cm phantom: 0.039 mSv/[mGy·cm] for children up to 4 months, 0.026 mSv/[mGy·cm] between 4 months and 1 year of age, and 0.018 mSv/[mGy·cm] between 1 year and 6 years of age[29].

### Statistics

Statistical analysis was performed with SPSS 17.0 software (SPSS, Chicago, IL, USA). Quantitative variables were described as means ± standard deviations, and categorical variables were given in frequencies or percentages. The subjective image quality scores were compared by using the Mann-Whitney U test. Interobserver agreement on grades of subjective image quality analysis was assessed by kappa statistics (κ>0.81, excellent agreement; κ = 0.61–0.80, good agreement). The independent *t* test was performed to analyze the differences between the two groups regarding image noise, SNR and CNR. Comparative analysis of the diagnostic performance between FBP and SAFIRE algorithm was obtained by non-parametric chi–square test. P<0.05 was considered statistically significant.

## Results

All 28 patients underwent successful low-dose DSCT angiography. The patient demographics are given in Table 1. Prospective ECG-triggered high-pitch spiral scan mode was used in 14 patients; whereas prospective ECG-triggering sequential scan mode was applied in the other 14 patients.

**Table 1:** Patient demographics, CT acquisition parameters and radiation dose estimates.

Male sex, no. of patients (of total)	16(28)
Age (months), mean±SD	13.25±13.05 (range: 2–48)
Weight (kg), mean±SD	8.14±3.16 (range: 4–16)
Heart rate during scan (bmp), mean±SD	119.86±16.21 (range: 89–149)
Tube voltage (kVp)	70
Tube current (mAs), mean±SD	95.71±16.87 (range: 70–130)
CTDIvol (mGy), mean±SD	0.47±0.27 (range: 0.20–1.14)
DLP (mGy·cm), mean±SD	6.11±2.70 (range: 3–16)
Effective radiation dose (mSv), mean±SD	0.30±0.13 (range: 0.16–0.78)

### Subjective image quality

**Figure 1:**
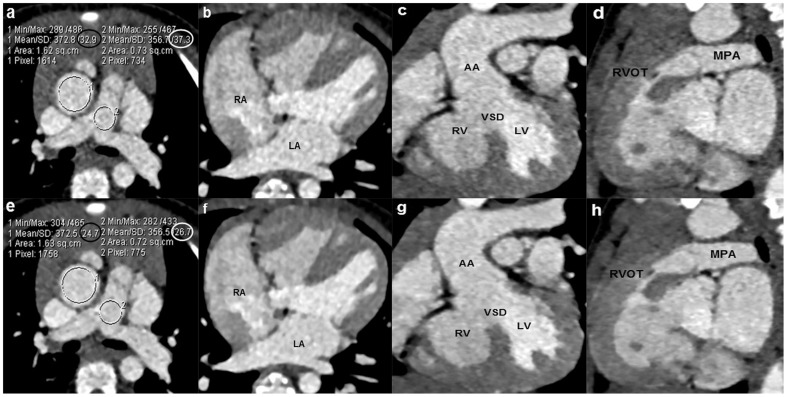
An 18-month boy with the diagnosis of Tetralogy of Fallot and atrial septal defect. The sequential DSCT angiography was performed with 70/rotation (effective radiation dose, 0.32 mSv). Axial images (a,e), multiplanar reformatted (MPR) images (b–d, f–h) using filtered back projection (FBP) algorithm (a–d) and sinogram affirmed iterative reconstruction (SAFIRE) algorithm (e–h) are shown. Image noise of the ascending aorta and pulmonary trunk which is expressed as the standard deviation (SD) of the attenuation (HU) in the regions of interest is significantly reduced in images reconstructed by SAFIRE (black and white circles in f) in contrast to FBP black and white circles in a). MPR images reconstructed with SAFIRE algorithm exhibit substantially reduced image noise and the improved image quality compared with images obtained with FBP. LA  =  left atrium, RA  =  right atrium, RV  =  right ventricle, LV  =  left ventricle, VSD =  ventricular septal defect, AA  =  ascending aorta, RVOT =  right ventricular outflow tract, MPA  =  main pulmonary artery.

**Figure 2:**
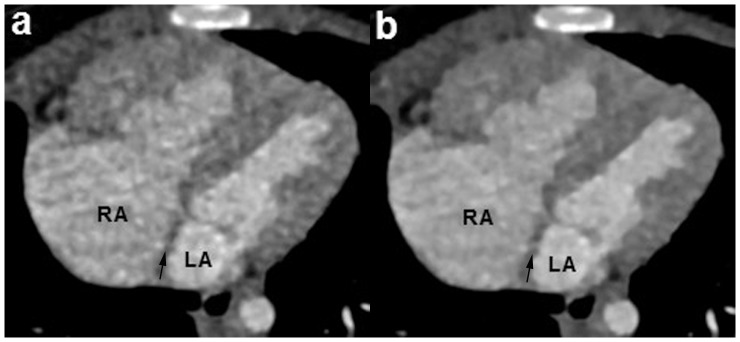
A 7-month girl with a small atrial septal defect. The sequential DSCT angiography was performed with 70/rotation (effective radiation dose, 0.21 mSv). Multiplanar reformatted (MPR) image reconstructed with sinogram affirmed iterative reconstruction (SAFIRE) algorithm (b) shows the small atrial septal defect (arrow) clearly. However, the lesion was missed on the MPR image using filtered back projection (FBP) algorithm (a). LA  =  left atrium, RA  =  right atrium.

The mean scores were significantly higher with SAFIRE algorithm than with FBP algorithm regarding to graininess, sharpness and overall image quality (Table 2). Diagnostic images (images graded 3 or more) obtained with the SAFIRE algorithm (28/28) were more than those with the FBP algorithm (23/28). Interobserver agreement was good and excellent for FBP (κ = 0.74) and SAFIRE (κ = 0.83) series, respectively. Representative cases are shown in Figure 1 and Figure 2.

**Table 2:** Subjective image quality assessment of filtered back projection (FBP) and sinogram affirmed iterative reconstruction algorithm (SAFIRE) series at 70 kVp dual-source CT (DSCT) angiography.

Subjective measure of image quality	Grade, mean±SD		P
	FBP	SAFIRE	
Graininess	2.82±0.77	3.89±0.74	<0.05
Sharpness	3.14±0.76	4.18±0.72	<0.05
Overall image quality	3.07±0.66	4.07±0.66	<0.05

### Objective image quality

**Figure 3:**
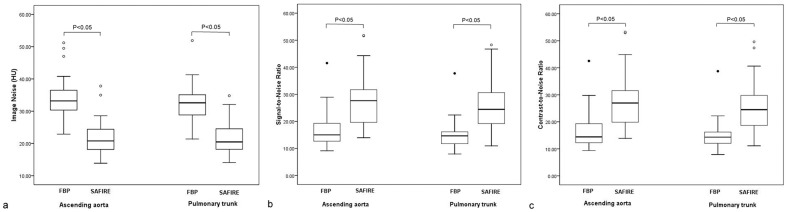
Box-and-whisker plots of objective analysis of image quality acquired with FBP and SAFIRE algorithms. Image noises of ascending aorta and pulmonary trunk were significantly lower with SAFIRE algorithm than with FBP algorithm (a). Signal-to-noise ratios and contrast-to-noise ratios of ascending aorta and pulmonary trunk were significantly higher with SAFIRE algorithm than with FBP algorithm (b,c). Center horizontal lines show median values and whiskers represent upper and lower quartiles. ○/•  = outliners.

The mean image noise in the ascending aorta and pulmonary trunk was significantly lower (t = 7.1 and 7.7 respectively, P<0.05) with SAFIRE algorithm than with FBP algorithm. The mean SNR (t = 4.7 and 5.1 respectively, P<0.05) and the mean CNR (t = 4.5 and 5.0 respectively, P<0.05) in the ascending aorta and pulmonary trunk were significantly higher with SAFIRE algorithm than with FBP algorithm. The details of objective image quality evaluation are shown in Table 3 and Figure 3.

**Table 3:** Image noise, signal-to-noise ratio (SNR) and contrast-to-noise ratio (CNR) in filtered back projection (FBP) and sinogram affirmed iterative reconstruction algorithm (SAFIRE) series at 70 kVp dual-source CT (DSCT) angiography.

Region of interest	Image noise (HU), mean±SD			SNR, mean±SD			CNR, mean±SD		
	FBP	SAFIRE	P	FBP	SAFIRE	P	FBP	SAFIRE	P
Ascending aorta	34.06±6.99	22.07±5.57	<0.05	17.16±7.11	28.33±10.28	<0.05	17.01±7.37	28.11±10.65	<0.05
Pulmonary trunk	32.85±5.78	21.55±5.23	<0.05	15.28±5.51	26.02±9.72	<0.05	15.14±5.65	25.80±9.78	<0.05

### Diagnostic accuracy

A total of 78 separate cardiovascular anomalies were confirmed by surgical and/or CCA findings. The details on separate cardiovascular abnormalities are shown in Table 4. There was no significant difference in the diagnostic accuracy between the FBP and SAFIRE algorithm (98.68% and 99.62%, respectively; χ^2^ = 1.793, P>0.05). Both FBP and SAFIRE series misdiagnosed one small atrial septal defect as normal. The FBP series failed to identify 3 cases of atrial septal defect, one ventricular septal defect, one pulmonary artery stenosis and one patent ductus arteriosus, while one small atrial septal defect was missed in SAFIRE series.

**Table 4:** Findings by filtered back projection (FBP) and sinogram affirmed iterative reconstruction algorithm (SAFIRE) series at 70 kVp dual-source CT (DSCT) angiography.

Cardiovascular deformities	FBP				SAFIRE				Surgical/CCA results
	TP	TN	FP	FN	TP	TN	FP	FN	
Atrial septal defect	5	19	1	3	7	19	1	1	8
Ventricular septal defect	12	15	0	1	13	15	0	0	13
Right ventricular outflow tract stenosis	3	25	0	0	3	25	0	0	3
Double outlet right ventricle	2	26	0	0	2	26	0	0	2
Pulmonary artery atresia	3	24	0	1	4	24	0	0	4
Pulmonary artery stenosis	3	25	0	0	3	25	0	0	3
Dilated pulmonary artery	3	25	0	0	3	25	0	0	3
Anomalous origin of pulmonary artery	1	27	0	0	1	27	0	0	1
Anomalous pulmonary venous return	2	26	0	0	2	26	0	0	2
Patent ductus arteriosus	8	19	0	1	9	19	0	0	9
Overriding aorta	5	23	0	0	5	23	0	0	5
Coarctation of the aorta	7	21	0	0	7	21	0	0	7
Interrupted aortic arch	1	27	0	0	1	27	0	0	1
Right aortic arch	4	24	0	0	4	24	0	0	4
Aortopulmonary window	1	27	0	0	1	27	0	0	1
Transposition of the great arteries	3	25	0	0	3	25	0	0	3
Major aortopulmonary collateral artery	3	25	0	0	3	25	0	0	3
Double superior vena cava	2	26	0	0	2	26	0	0	2
Coronary artery anomaly	4	24	0	0	4	24	0	0	4
Total	72	453	1	6	77	453	1	1	78

TP, true positive detection; TN, true negative detection; FP, false positive detection; FN, false negative detection.

### Radiation dose

Radiation dose of 70 kVp DSCT angiography is given in Table 1. The mean CTDIvol was 0.47±0.27 mGy. The mean DLP was 6.11±2.70 mGy·cm, corresponding to a mean estimated effective dose of 0.30±0.13 mSv.

The CTDIvol (t = 6.918, P<0.05), DLP (t = 3.625, P<0.05) and the effective radiation dose (t = 2.147, P<0.05) of the high-pitch group were lower than those of the sequential scanning group. The mean CTDIvol of the high-pitch group and the sequential scanning group was 0.26±0.05 mGy and 0.69±0.23 mGy. The mean DLP of the two groups was 4.57±1.22 mGy•cm and 7.64±2.92 mGy•cm, resulting in a mean estimated effective dose of 0.25±0.07 mSv and 0.35±0.16 mSv.

## Discussion

The results from our study indicate the feasibility of 70 kVp-tube-voltage DSCT angiography in children with CHD. By applying SAFIRE algorithm with 70 kVp-tube-voltage CT examination, the image quality and diagnostic confidence were maintained with significantly image noise reduction and low radiation dose of 0.30±0.13 mSv.

### 70 kVp-tube-voltage technique

Lowering the tube voltage has the advantage of higher vascular enhancement with a reduced radiation dose. Iodine attenuation increases as the tube voltage decreases because the mean photon energy moves closer to the k-absorption edge of iodine. With the introduction of a new x-ray tube, voltage as low as 70 kVp can now be applied. As the radiation dose is proportional to the square of the tube voltage, the 70 kVp-tube-voltage technique has the potential to further reduce the radiation dose.

Besides 70 kVp-tube-voltage technique, several dose-saving strategies were applied in our study including the adapted tube current to body weight, the prospective ECG-triggering sequential mode and high-pitch mode. Our previous studies [8] demonstrated that the high-pitch mode, in comparison with the sequential mode, further lowers the radiation dose. In this study, the effective dose of high-pitch group and sequential scanning group was 0.25±0.07 mSv and 0.35±0.16 mSv, respectively. However, the high-pitch mode is not the only choice. Because when the indication of DSCT angiography is to evaluate the coronary artery abnormalities, the sequential mode would be recommended as the relatively poorer performance of the high-pitch mode on demonstrating coronary arteries in patients with irregular heart rate.

The downside of the 70 kVp-tube-voltage CT scanning is the increased image noise which may impair diagnostic confidence. To counterbalance the increased image noise, a higher tube current time product compared with our previous study [7], [8] was used with the combination application of an iterative reconstruction algorithm.

### Sinogram affirmed iterative reconstruction algorithm (SAFIRE)

Conventional CT image reconstruction such as FBP comprises a trade-off between sharpness and noise. Sharpness can only be increased at the expense of higher image noise, or vice versa, noise can only be reduced by decreasing sharpness. This trade-off limits the minimum radiation dose required for a specific diagnostic task since the lower radiation dose is associated with the increased image noise [22], [25], [27].

SAFIRE, as one recently introduced IR algorithm, represents an iterative optimization process that overcomes the constraint of FBP by using a noise modeling technique supported by the raw data with the aim of decoupling the relationship between sharpness and image noise [22]-[27]. There are two different correction loops in SAFIRE algorithm. The first loop occurs in the raw data space. After an initial reconstruction used FBP, the detected deviations are corrected by the repeated reconstruction with FBP to generate an updated image. A dynamic raw data-based noise modeling technique is used allowing for noise reduction without noticeable loss of sharpness. The second loop occurs in image space, where the noise is estimated and subtracted from the current dataset. The corrected image is compared with the original data leading to an update image, and added to the previous dataset before the next iteration is performed. This process is repeated a number of times, until the desired image is achieved. [23]–[25], [27]

The benefits of SAFIRE algorithm for various clinical applications have been shown in previous studies [22]–[27]. In our study, the graininess, sharpness and overall image quality were significantly improved with SAFIRE, in the meanwhile, SAFIRE algorithm yielded a lower image noise and higher SNR and CNR than FBP algorithm. The diagnostic accuracy between FBP and SAFIRE algorithm was not significantly different by Han BK et al. [24], however, there was an incremental improvement in the detection of small atrial septal defect, ventricular septal defect, pulmonary artery stenosis and patent ductus arteriosus with SAFIRE algorithm compared to FBP from our results.

Our study has some limitations. First, a relatively small group of patients were enrolled, a large cohort of population are needed in the future study. Second, the 5 levels of iteration presets of SAFIRE (strength 1–5) associated with its ability of noise reduction were not compared. Third, the potential dose reduction of SAFIRE with 70 kVp tube voltage was not assessed. Last, the amount of contrast material was not considered in this study. Because tube voltage reduction increases the attenuation of iodinated contrast material, it may be possible to reduce the amount of contrast material administered with 70 kVp DSCT angiography.

In conclusion, the application of SAFIRE algorithm with 70 kVp DSCT angiography significantly reduces the image noise and improves the image quality in comparison with FBP algorithm. The combination of low-tube-voltage DSCT angiography with the SAFIRE algorithm in children with CHD is recommended.
